# University students’ views on the impact of Instagram on mental wellbeing: a qualitative study

**DOI:** 10.1186/s40359-022-00743-6

**Published:** 2022-02-28

**Authors:** Laura Moreton, Sheila Greenfield

**Affiliations:** 1 College of Medical and Dental Sciences, University of Birmingham, Birmingham, B15 2TT UK; 2 Institute of Applied Health Research, University of Birmingham, Birmingham, B15 2TT UK

**Keywords:** Instagram, University, Students, Mental wellbeing, Qualitative

## Abstract

**Background:**

In the UK, undergraduate students are considered highly susceptible to mental ill-health, with current figures indicating a 94% increase in the demand for university counselling services in the last five years alone. Whilst the cause of this increase is currently undetermined, current evidence speculates that social media may be a contributing factor. Recent quantitative literature has determined that Instagram can negatively impact mental wellbeing. However, limited studies have been conducted among undergraduate student students, a population with the highest proportion of Instagram use by age, compared to the general public. Furthermore, no current research has qualitatively explored how and why Instagram has an impact on mental wellbeing. This study therefore aimed to identify UK university students’ understanding of the term ‘mental wellbeing’ and explore university students’ views on the impact of Instagram on their mental wellbeing.

**Methods:**

16 undergraduate students (56.3% female, mean age 19.8 years, age range 18–23 years) who were fluent in English and used Instagram took part in semi-structured interviews. Participants were excluded if they no longer used Instagram or were unable to give written informed consent. Thematic analysis was utilised to identify codes and themes within the dataset.

**Results:**

Five key themes were identified; knowledge of mental wellbeing, social connectivity, the Instagram ideal, social acceptance through quantitative data and cyberbullying. Students had a basic understanding of the term mental wellbeing and correctly associated productivity and accepting life’s adversities with the term. However, students often misinterpreted happiness and good mental health as a state of wellbeing. Whilst students perceived Instagram as positive for the development and maintenance of friendships, they also believed Instagram negatively impacted their wellbeing through the presentation of ideals, the presence of cyberbullying and the search for social acceptance.

**Conclusions:**

This research reveals multiple implications for Instagram use on the mental wellbeing of undergraduate students. It is therefore essential for university counselling services to address Instagram use in consultations with students. Further research exploring the extent of cyberbullying on Instagram and the effects of presenting an idealistic life on wellbeing is necessary.

## Background

The World Health Organisation defines mental health as “a state of well-being in which the individual realises his or her own abilities, can cope with the normal stresses of life, can work productively and fruitfully, and is able to make a contribution to his or her community” [1]. Those with poor mental wellbeing are at an increased risk of mental illness development and furthermore, have a reduced life expectancy [2, 3].

In the UK, one in four individuals suffer from a mental illness in their lifetime [4]; the majority of which arise before the age of 25 years [5]. 83% of undergraduate students in the UK are between the ages of 16–24 years and are hence considered to be highly vulnerable to mental ill-health [6, 7]. Figures indicate that the prevalence of mental illness among undergraduate students has significantly increased in the last 10 years, demonstrated by a 94% increase in the demand for university counselling services in the last five years alone [8]. Whilst the cause of this increase is currently undetermined, there is speculation in current literature that the advent of social media (SM) may be a contributing factor with a further emphasis on the impact of image-centric SM platforms such as Instagram [9–20].

In the UK, 91% of 16–24 year-olds have at least one SM account and spend three hours per day on average on SM sites [21, 22]. Whilst SM affords users the opportunity to disseminate information, increase one’s social capital and communicate with a global audience, current research indicates that SM may have a negative impact on one’s mental wellbeing [11, 20, 21, 23, 24]. Recent research has focused on image centric SM platforms, such as Instagram, as they may be particularly harmful for the body satisfaction, self-esteem and the psychological wellbeing of its users when compared to text based platforms [25, 26]. It is recognised that the visual nature of image-centric SM platforms allows greater opportunities for upward social comparisons (comparisons to others believed to be superior than oneself) which may drive the body dissatisfaction and reduced self-esteem of its users [25, 27, 28].

Fardouly et al., determined that users of visually-focused SM platforms, such as YouTube and Instagram, had greater concerns with their body image compared to those who did not use SM [29]. Furthermore, Engeln et al. concluded that undergraduate women who used Instagram had greater body dissatisfaction and increased negative affect compared to those who used Facebook [25]. Additionally, Boursier et al. established that users with higher levels of body surveillance and appearance anxiety were more likely to post self-images on SM with the aim of achieving positive feedback from peers to gain confidence [30, 31]. Furthermore, Gioia et al. concluded that male adolescents were more likely than females to edit photos and change their body image in order to obtain an ideal appearance for SM [32]. This thus indicates that photo-based SM platforms may be more detrimental to the mental wellbeing of its users compared to text-based platforms due to body dissatisfaction, social comparison, social acceptance and internalisation of the ideal.

Founded in 2010, Instagram is a photo-sharing SM platform allowing users to post photographs and videos onto their profiles [33]. Users interact with one another using ‘likes’ and ‘comments’ and can follow an unlimited number of people [15]. Public engagement with an individual’s post can be increased through the use of captions, geotags and hashtags (#) [15].With approximately 23 million users in the UK alone and 52 million images posted per day, Instagram is the second most popular SM platform after Facebook [29, 34–36]. In a 2017 survey conducted by the Youth Health Movement, 1,500 14–24 year olds ranked Instagram as the worst SM platform for their mental well-being, however, it was not determined why this was the case [21]. A recent systematic review concluded that increased Instagram use was associated with greater social comparison, body dissatisfaction and eating disorders amongst its users which may explain the detrimental impact of Instagram on mental wellbeing [27].

Current research indicates that Instagram affects the body satisfaction of its users [16, 29, 37–41]. Tiggemann et al. concluded that Instagram photos of thin women increased body dissatisfaction and appearance comparison among female students compared to photos of average-sized women [37, 38]. Furthermore Cohen et al. report increased body surveillance amongst female Instagram users compared to non-users [39]. Moreover, a qualitative study by Chatzopoulou et al. involving young men engaging with the Instagram fitness community, discovered that those with lower self-esteem were greater motivated to change their body in order to ascertain the ideal body displayed on Instagram [40]. Additionally, users felt discouraged to post self-images if they felt their body did not meet the proposed ideal [40]. A significant association has also been ascertained between the time spent editing photos following exposure to photos of thin women on Instagram and the desire to look better than in real life, indicating an internalisation of the thin ideal [38].

Further to this, research indicates a correlation between Instagram usage and the presence of eating disorder symptoms [15, 17, 29, 42]. Griffiths et al. have established that the use of image-centric SM platforms (such as Instagram) significantly increase the likelihood of eating disorder symptoms among sexual minority men [17]. Additionally, Turner et al. have also found an association between Instagram usage and orthorexia nervosa, a relatively new concept in which individuals are obsessed with healthy eating and is characterised by ‘food anxiety and dietary restrictions’ leading to malnutrition [15, 43]. Further research has also indicated that in pre-adolescent females, increased Instagram usage is significantly associated with an increase in clinically determined eating disorder symptoms [42].

Current research has generally focused on adolescents’ Instagram use with only a limited number of quantitative studies being conducted among undergraduate students, a population with the highest proportion of Instagram use by age, compared to the general public [37, 38, 44]. At present, no research has quantitatively determined the relationship between Instagram and mental wellbeing in UK university students. Moreover, results from current international literature are predominantly determined utilising quantitative methodological approaches, utilising various psychiatric and psychological scales [9–21, 24, 29, 36–39, 41, 42]. To the best of the authors’ knowledge, no research has qualitatively explored how and why Instagram has an impact on mental wellbeing, an approach that is likely to reveal deeper insights into participants’ personal experiences with Instagram and which could allow for the discovery of new concepts previously unknown to researchers [45, 46]. Hence, this study aimed to identify UK university students’ understanding of the term ‘mental wellbeing’ and explore university students’ views on the impact of Instagram on their mental wellbeing.

## Methods

### Participants and setting

Participants were students currently undertaking an undergraduate degree qualification at the University of Birmingham, a UK based, campus university with 22,412 undergraduate students [47, 48]. Students who were fluent in English and used Instagram were eligible for participation. Participants were excluded if they no longer used Instagram or if they were unable to give written informed consent.

### Recruitment

Convenience sampling was utilised to recruit participants [49]. Posters advertising the study were displayed in the main university library and various university faculty buildings. Each poster contained information outlining the study including the study title, the eligibility criteria, advertisement of the study incentive (a £10 amazon voucher) and the researcher’s contact details.

Students who expressed an interest in the study were subsequently emailed a participant information leaflet and an eligibility questionnaire containing questions on their level of education, English speaking capability, Instagram usage and gender. Information from the eligibility questionnaire was then utilised to determine whether participants fulfilled the eligibility criteria. Furthermore, as male views are vastly under-represented in current social media literature, the eligibility questionnaire allowed purposive sampling to ensure equal representation of each gender to be selected for participation [50]. Two students who were willing to participate had deleted Instagram due to its negative effect on their wellbeing and therefore, had to be excluded. For eligible students, the first ten females and males to return the eligibility questionnaire were recruited. No new analytical themes were identified following interviews with 16 participants indicating that data saturation was reached; hence recruitment ceased [51].

### Data collection

Individual, face-to-face, semi-structured interviews were deemed the most appropriate qualitative methodology due to the potential emergence of sensitive content [52]. Interviews were undertaken in February 2020 in a private study room of the university’s library and lasted between 20 and 55 min with a mean length of 34 min. LM, a female medical student intercalating in Public Health and Population Sciences who uses Instagram, conducted each interview. Participants had no prior acquaintance with LM before interview commencement. Written informed consent was obtained before each interview. Each interview was guided by a self-constructed topic guide with support from existing quantitative literature on social media and mental health. Each question was designed to be open-ended, allowing for in-depth exploration of each topic and furthermore, reduced interview bias [52, 53]. The topic guide was refined following a pilot interview with additional questions included to further address the research aims. Data from the pilot interview was not included in the final analysis of results. Following each interview, participants received a £10 amazon voucher as a token of gratitude for their time. Field notes, including information regarding participant behaviours and body language, were completed following interviews to support data analysis [54]. No repeat interviews were completed.

### Data analysis

Interviews were audio-recorded and manually transcribed by LM. Each transcript was thematically analysed, utilising Braun and Clarke’s six step framework [55, 56]. Codes and themes were identified inductively from the dataset [55, 56]. Transcripts were re-read thrice to attain data familiarisation [55, 56]. A rapid analysis of themes then ensued to achieve full immersion within the data [56, 57]. Codes were then generated inductively from each transcript. Each code was subsequently inputted into a Microsoft Excel spreadsheet with codes for each participant tabulated, allowing for the comparison of codes according to gender and for the identification of deviant cases [58]. With the aid of spider diagrams and the Excel spreadsheet, codes were organised into categories and initial themes were developed. No further codes, categories or themes arose from the final three transcripts, confirming that data saturation had been achieved [51].

To address the trustworthiness of this research, analytic triangulation was completed [59]. Two researchers (LM and SG) independently analysed each transcript and met to discuss, and further refine the identified themes. Furthermore, each participant was emailed a copy of the themes generated from their transcript, allowing for member validation to occur [60]. 14 students (87.5% of participants) responded, indicating that the themes identified were accurate interpretations of their views, validating the research results [60].

LM worked reflexively throughout the process of data collection and analysis, ensuring that her personal use of Instagram did not affect the study results or introduce bias [61, 62]. Utilising a reflexive journal LM recorded her preconceived perceptions regarding Instagram and noted how her views had changed following each interview.

## Results

**Fig. 1:**
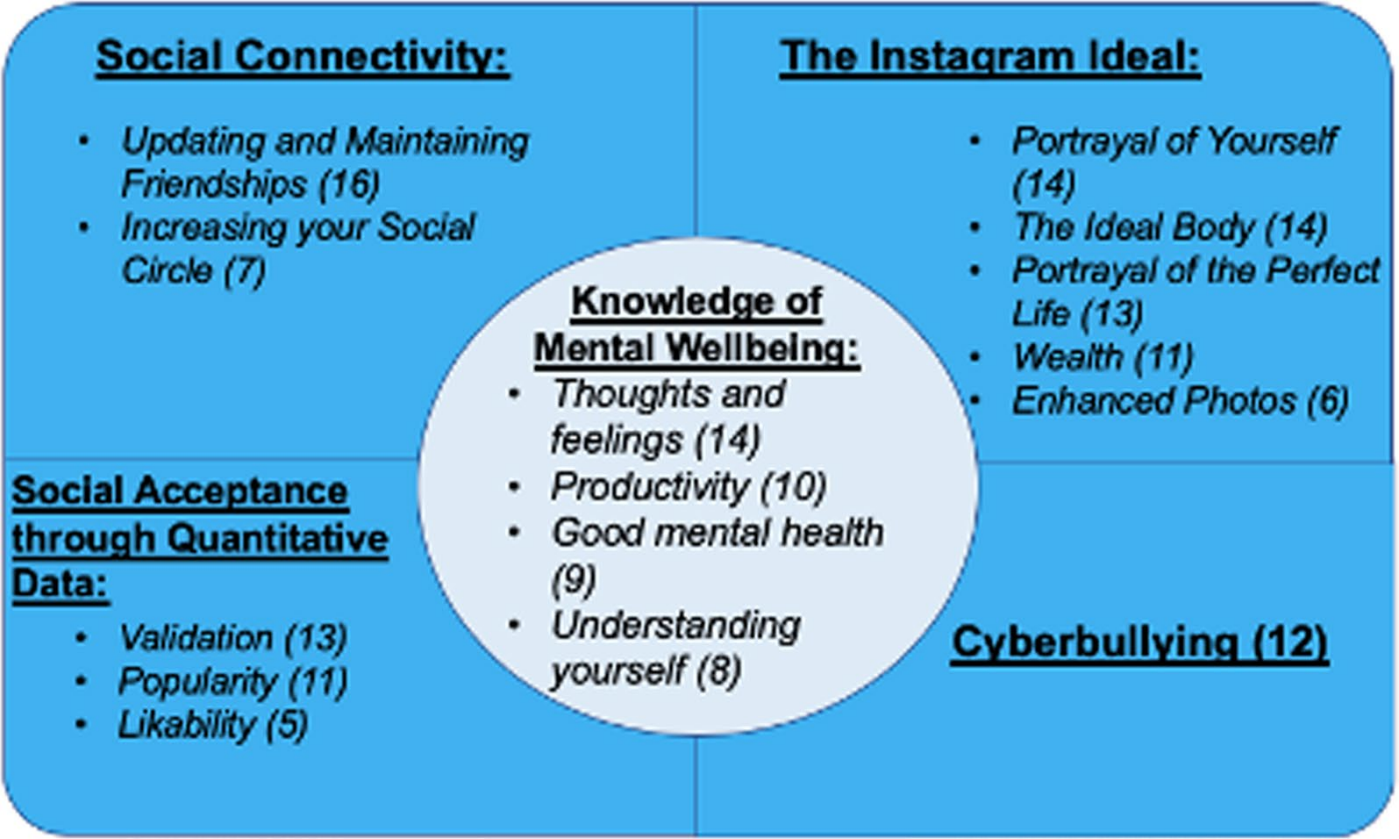
A diagram illustrating the identified themes and associated subthemes. Each section of the diagram represents a key theme. The bullet points in each section demonstrate the subthemes for each theme. Each number indicates the prevalence amongst the dataset

A total of 16 students were interviewed with a mean age of 19.8 years (range 18–23 years). 56.3% of participants were female (Table 1). Pseudonyms are used in place of participant names to ensure confidentiality. Gender did not influence the observed themes. Five themes and associated subthemes arose from the dataset; knowledge of mental wellbeing, social connectivity, the Instagram ideal, social acceptance through quantitative data and cyberbullying. To aid readability, each theme and associated subtheme is presented in order of prevalence amongst the dataset. Figure 1 depicts each theme with their associated subthemes.

**Table 1:** Participant characteristics

Participant’s pseudonym	Age (years)	Gender	Self-estimated Instagram usage (in minutes/day)
Harry	20	Male	30
Grace	20	Female	60
Mia	19	Female	30
James	20	Male	20
Harriet	20	Female	20
George	20	Male	60
Isabelle	19	Female	20
Alice	18	Female	60
Charlotte	23	Female	30
Sophia	18	Female	20
Ethan	20	Male	100
Noah	21	Male	90
Charlie	18	Male	90
Amelia	20	Female	90
Marie	20	Female	40
Oliver	21	Male	30

### Knowledge of mental wellbeing

Four subthemes were identified regarding students’ understanding of mental wellbeing; mental wellbeing as thoughts and feelings, productivity, good mental health and understanding yourself.

#### Thoughts and feelings

The majority of students believed one’s thoughts and emotions determined mental wellbeing and further, perceived positive emotions as the foundation for good mental wellbeing.

Harry: “it’s like how we’re thinking what are our emotions…are we feeling good or not”

Grace: “It is like obviously feeling positive and happy”

Furthermore, eight students believed mental wellbeing was fluctuating in nature due to changes in emotions and hence varied day to day.

Mia: “it’s like a wave or like a rollercoaster…”

James: “it may just be something’s made you bit down one day and therefore your mental wellbeing won’t be as good as it would another day”

#### Productivity

Most students associated good mental wellbeing with being productive and the ability to do work.

Harriet: “being able to kind of participate in society happily…like productively and being able to work”

George: “I think just being able to…cope with one’s amount of like workload”

#### Good mental health

Students generally perceived mental wellbeing as good mental health and often assumed mental illness was a poor state of wellbeing.

Isabelle: “mental wellbeing is a fluctuating thing…most people at points in their life experience mental health problems”

Alice: “everybody has a mental wellbeing…I mean obviously when things aren’t quite right and there is a mental illness…”

#### Understanding yourself

Half of the students thought mental wellbeing related to accepting your surroundings and dealing with hardships in life. Moreover, students believed being yourself underpinned mental wellbeing.

Charlotte: “I think if…you have a good understanding of what’s going on around you then fair your mental wellbeing is good but maybe if you’re…ignoring something to have happiness…that’s not quite mental wellbeing”

Sophia: “it’s about you…being yourself in society…so like being yourself that’s what I mean”

Ethan: “you can be unhappy with life but you can still be mentally well if you accept where you are and understand yourself…realise your situation…just sort of accept it”

### Social connectivity

Instagram was perceived to be positive for mental wellbeing by allowing connection and communication with others. Two subthemes were identified; updating and maintaining friendships and increasing your social circle.

#### Updating and maintaining friendships

All students thought Instagram was a great platform for updating friends, arranging meetups and believed it allowed users to maintain friendships.

Sophia: “catch up with friends and stuff…so whenever I wanna meet up at a restaurant or I wanna go shopping I’d often just use Instagram”

Ethan: “mainly to keep in contact with friends or see what they’re up to…cause…that’s what most people erm update their social circle through”

#### Increasing your social circle

Half of the students mentioned that Instagram allowed them to increase their social circle and introduce them to new people, creating further friendships.

Noah: “maybe with the influence of social media because everyone can talk around the world now and it’s like all so inter-connected”

Charlotte: “I get to see who other people are connected to and then oh actually it turns out I know that person and like I can build my network as well”

### The Instagram ideal

All students stated that Instagram was a platform utilised to show off to others and hence creates an unrealistic ideal to live up to. Five subthemes were identified; portrayal of yourself, the ideal body, portrayal of the perfect life, wealth and enhanced photographs.

#### Portrayal of yourself

Nearly all students noted that they would only put the best version of themselves online and even remarked that they would delete photos if they were not looking their best.

Charlie: “…I’m quite selective with what I post I wouldn’t post a BAD image on Instagram…”

Alice: “I’ve posted something and deleted it a couple times …if I don’t think that I look my best then I’ll 100% take it off”

#### The ideal body

The majority of the students believed Instagram distorted their idea of a healthy body shape, with most pictures presenting an ‘ideal body’ type.

Isabelle: “it seems like it would be normal to hate your body because of all these bodies on Instagram…as this one thing and you had to look this certain way”

Amelia: “I was like “oh she’s got look at her she’s Kim K’s body”…I’d think but my body’s not like that…so I used to literally do a lot like be exercising as much and even that wasn’t the result because I was not gonna look like a surgical body”.

#### Portrayal of the perfect life

Almost all students believed people on Instagram only show the best parts of life and hence Instagram distorts their sense of reality.

Marie: “people do only post the positives of their life…just like will post an Instagram story of them on holiday an Instagram post of them there then here…”

Oliver: “…they only post the things that is glamourous and actually they have like a family problems or that but they didn’t show off to the public they just show the good stuff”

#### Wealth

Over half of the students perceived wealth to be rife on Instagram with branded clothes, destination holidays and expensive possessions being the forefront of attention.

Noah: “these people don’t have a house but they’ve got all these nice…branded clothes…if I get a mortgage I’m not gonna flaunt that but you’re gonna flaunt your clothes"

Oliver: “they buy some expensive gadgets or equipment or some expensive clothing so they will post it on Instagram”

#### Enhanced photographs

A few students also noted that the use of editing software such as ‘Facetune’ and ‘Photoshop’ further added to their altered sense of reality by creating an unrealistic appearance to live up to.

Harriet: “with photoshopped photos or even the filters… that’s not what they look like in real life…you’re gonna be trying to look like that and…no one looks like that…that definitely hurts a lot of people”

Harry: “you can edit the pictures like they have many…different ways to edit pictures…I just feel like the pictures become…not that natural anymore”

### Social acceptance through quantitative data

Almost all students believed social acceptance was demonstrated through ‘likes’ and ‘followers’, with higher numbers associated with greater social acceptance. Three subthemes were identified; validation, popularity and likability.

#### Validation

The majority of the students assumed that Instagram ‘likes’ and ‘comments’ represented friendship. Others also believed ‘likes’ provided evidence of an attractive appearance in photos.

James: “I guess if a hundred people like it then you’re like oh well must be a nice photo like a good looking photo…you get a feel of satisfaction when you post something…”

Marie: “people to like and comment on it sort of confirms that you’re friends”

#### Popularity

Most students also believed quantitative data on Instagram represented popularity as those with large numbers of Instagram ‘likes’ or ‘followers’ were believed to have more friends.

Charlie: “I suppose if someone’s posting with all their friends and then that post had good reactions then that can make you feel unpopular if you’re not included in that post”

Grace: “people comment on it or like likes…it makes me feel happy which is silly but it does it makes me feel popular”

#### Likeability

Some students believed that ‘likes’ represented one’s likeability. Those with few ‘likes’ were deemed less ‘likeable’ in real life.

Charlotte: “I think when you see someone who gets likes… I’m like oh people LIKE them”

Sophia: “they’d be getting like…300 likes but then I’m…getting like around 50… I thought that I was like erm not really a likeable person”

### Cyberbullying

Most students were nervous to post on Instagram due to fear of judgement from others. A further nine students perceived cyberbullying and ‘internet trolls’ on Instagram as a contributing factor to poor mental wellbeing.

Marie: “erm I don’t post on Instagram that much cause sometimes…I do feel like I’ll be judged for it”

Alice: “people used to bully me because they would say I have a big forehead… I used to get like a couple comments on my Instagram…”

George: “there are some people who make troll accounts and bash other people”

## Discussion

### Main findings

This research reveals that the students interviewed had a basic understanding of the term mental wellbeing. Furthermore, whilst students remarked that Instagram was positive for the development and maintenance of friendships, students also believed Instagram had multiple implications for mental wellbeing, namely the presentation of ideals, the search for social acceptance and cyberbullying.

Whilst many students accurately perceived mental wellbeing as the ability to cope, accept life stressors and productivity, students often misinterpreted happiness and having good mental health as a state of mental wellbeing, a finding consistent with previous research [63]. Notably, some students perceived mental wellbeing and mental illness to be on the opposite end of the same scale, highlighting confusion amongst students regarding the difference between the two terms.

With regards to Instagram, most students indicated that they would only present the best version of themselves on the platform in order to show off to their peers. Previous research reports similar findings in which students select the best ‘selfies’ and furthermore, edit their photos in order to look better before posting photos on Instagram [32, 38]. This behaviour was observed among students who desired social acceptance from peers, with the perception that an increase in Instagram ‘likes’ was associated with validation, popularity and likeability. The desire for validation and the perception of popularity and likeability associated with ‘likes’ is consistent with previous research identifying validation seeking behaviours amongst social media users [14, 16, 30–32, 40, 64].

In line with previous research, students reported the presence of beauty ideals on Instagram with most students suggesting the presentation of ‘perfect’ bodies creates an unrealistic ideal to live up to [29, 37–40]. Furthermore, 37.5% of students reported that the use of editing software by celebrities and ‘influencers’ further manipulated their perception of natural beauty and noted the potential harm this could have on their mental wellbeing due to upward comparison [25, 27, 28]. This research therefore adds to the body of evidence indicating an internalisation of the thin ideal following Instagram, and wider social media use and perhaps explains previous results indicating a reduction in body satisfaction following the use of Instagram [29, 37–40].

This research extends the evidence provided by previous literature on the presentation of ‘ideals’ on Instagram. Students conveyed that Instagram portrays an idealistic, perfect life and often felt jealous of those who they perceived to have a better life than theirs. Students, however, also remarked that the presentation of life on Instagram was fictitious with most only showing the best parts of life and concealing negative aspects of life from their social media followers. Furthermore, students perceived people on Instagram to be highly wealthy due to the presentation of branded clothing, expensive gadgets and expensive destination holidays and as such believed they were poorer in comparison. The perceived appearance of an idealistic life and the perception of wealth has, as of yet, not been discussed in UK-based Instagram research; hence this research widens the current knowledge base regarding the presentation of ideals on Instagram.

Students also reported that cyberbullying and judgement from others on Instagram were major contributors to poor mental wellbeing with 56.3% of participants having witnessed or received online hate via the platform. Recent research indicates that increased time spent on Instagram is positively associated with cyberbullying and victimisation [26]. Research on generalised SM use has indicated that cyberbullying victims are at significantly increased risk of depressive symptoms and social anxiety [65]; hence Instagram may have a more profound effect on the mental wellbeing of its users than previous literature suggests.

### Recommendations for policy makers and future research

It is therefore essential for universities to address Instagram use amongst their students and furthermore, offer guidance and support to those affected. Counselling services within universities should be educated on the potential effects of Instagram amongst the undergraduate student population and as such, should address students’ concerns regarding social media. Future research is required to determine the extent to which cyberbullying via Instagram effects the wellbeing of students and additionally, should address whether the idealistic life portrayed on Instagram effects the life satisfaction of its users.

### Strengths and limitations

This is the first study to qualitatively explore the impact of Instagram on the mental wellbeing of UK university students. The use of interviews allowed for new concepts to be discovered and explored in depth. A further strength of this study was the high proportion of men interviewed, a population usually under-represented in SM research. The methodology of this research provides multiple strengths; the use of one researcher to conduct all interviews, analytic triangulation with an experienced researcher, the use of member validation and furthermore, the fact that data saturation was achieved ensures the consistency, trustworthiness and credibility of this research [51, 59, 60].

This research however does have limitations. The study was advertised as exploring the impact of Instagram on mental wellbeing; hence students with more extreme views may have volunteered to participate. Moreover, two students who no longer used Instagram due to its negative effects on their wellbeing were willing to participate yet had to be excluded as they did not meet the inclusion criteria; hence, the results may understate the effect of Instagram on mental wellbeing. A further limitation of this study is the lack of further data collected about each student’s characteristics; information was not obtained regarding their undergraduate degree subject, their mental wellbeing status, what they used Instagram for and whether their Instagram profile was a public or private account. Knowledge of these factors would have allowed further analysis to ascertain whether certain individual characteristics yielded specific themes amongst the data. Additionally, students’ self-estimated time spent on Instagram was recorded. This is a subjective measurement and is susceptible to recall bias, with many students underestimating their time spent on social media [66]. Therefore, we were unable to accurately draw conclusions about whether the amount of time spent on Instagram affected the themes portrayed.

## Conclusion

This is the first UK-based study to qualitatively examine the impact of Instagram on students’ mental wellbeing. Whilst findings corroborate previous international, quantitative research suggesting the internalisation of beauty ideals and validation seeking behaviour among individuals using Instagram, this research goes beyond current evidence, revealing further implications for mental wellbeing with Instagram use. It is therefore necessary for universities to address Instagram use amongst their most vulnerable students and offer guidance and support to those affected.

## Data Availability

The datasets generated and analysed during the current study are not publicly available due to confidentiality and privacy related issues but are available from the corresponding author on reasonable request.

## Abbreviation

SM: Social media
